# When a Negative Experience Sticks With You: Does the Revised Outcome Debriefing Counteract the Consequences of Experimental Ostracism in Psychological Research?

**DOI:** 10.1177/15562646241227065

**Published:** 2024-01-23

**Authors:** Stefanie Miketta, Malte Friese

**Affiliations:** 1 Department of Psychology, University of Hagen, Hagen, Germany; 2 Department of Psychology, 9379 Saarland University, Saarbruecken, Germany

**Keywords:** debriefing, research ethics, perseverance effect, continued influence effect, ostracism, cyberball

## Abstract

For research purposes, it is generally accepted that experimental ostracism manipulations can lead to a reduction of participants’ well-being. To eventually restore participants’ well-being, researchers rely on post-experimental debriefings that discredit prior deception. However, evidence suggests that discredited beliefs can persevere. The present research investigates whether a potent debriefing procedure restores participants’ well-being after an experimentally induced ostracism experience. In two studies, participants were either excluded or included in a Cyberball game, indicated their well-being, and were debriefed. In two additional conditions, participants were debriefed before indicating their well-being. Ostracism compared to inclusion led to decreased positive and increased negative mood. The debriefing did not counteract this effect (Studies 1 & 2). Unwanted aftereffects of the manipulation persevered for more than one day after the experimental session (Study 2). These findings question the effectiveness of debriefings and raise issues about research ethics.

In their *Ethical Principles of Psychologists and Code of Conduct*, the American Psychological Association (2017) deals with the question of inflicting harm on participants and sets a clear standard: “Psychologists [need to] take reasonable steps to avoid harming their … research participants … and to minimize harm where it is foreseeable and unavoidable” (p. 6). Starting decades ago, researches have thought of two different strategies on how to take those ‘reasonable steps’: (1) the abandonment of any harmful manipulations in psychological studies (e.g., Baumrind, 1964), and (2) the employment of precautions such as recruiting only psychologically stable individuals as participants (e.g., Kelman, 1967; Walster et al., 1967).

In social psychological research, these approaches have not been widely adopted: Manipulations that can lower participants’ well-being are being used and careful a priori screening of participants is usually not mentioned in the respective publications.

Historically, there have been vivid discussions regarding whether and how research can still be ethical in this context (e.g., Adair et al., 1983; Elms, 1975; Tesch, 1977; West & Gunn, 1978). Following Milgram's (1964) justification of his obedience studies, the idea of justifying the use of potentially harmful manipulations (even those that are not in accordance with the APA guidelines) through post-experimental debriefing became common practice (e.g., Adair et al., 1985; Sharpe & Faye, 2009; Smith & Richardson, 1983; Sommers & Miller, 2013; Tesch, 1977; Walster et al., 1967).

However, formal evidence for the effectiveness of debriefings is scarce (e.g., Sharpe & Faye, 2009; Tesch, 1977). The few existing studies conclude that debriefings are not necessarily effective (McFarland et al., 2007; Miketta & Friese, 2019; Oczak & Niedźwieńska, 2007; Ross et al., 1975; Silverman et al., 1970; Walster et al., 1967). As a result, several researchers made efforts to develop more effective debriefing procedures. For example, McFarland and colleagues (2007) compared a Standard Outcome Debriefing*,* which informed participants about the false feedback they had received, with their newly created Revised Outcome Debriefing that additionally informed participants about the fact that the ostensible social perceptiveness test that they took was not real either (and therefore invalid). This Revised Outcome Debriefing proved to be more effective in eliminating the effects of the manipulation than the Standard Outcome Debriefing.

The research by McFarland and colleagues suggests that the Revised Outcome Debriefing is the most potent type of debriefing and able to undo the effects of falsely given information. Doubts remain, however, if this conclusion extends to highly threatening experimental manipulations. In the work by McFarland et al. (2007), participants received false feedback about an ostensible social perceptiveness task in which they had to distinguish fake suicide notes from real ones. In the negative feedback condition, participants then received the feedback that they had performed poorly at distinguishing the suicide notes. However, in contrast to some widely used experimental manipulations in social psychology that are known to cause detrimental effects on well-being (e.g., Hartgerink et al., 2015; Leary et al., 2009), there is no empirical evidence for detrimental effects caused by the suicide note manipulation used by McFarland and colleagues. Furthermore, the ability to reliably distinguish real from fake suicide notes is not commonly considered a core competence relevant for one's personal identity.

Being included in social groups, by contrast, is a fundamental need (Baumeister & Leary, 1995; Smith et al., 1999) that has even been argued to be crucial for survival (Caporael, 1997; van Beest & Williams, 2006). It should thus not come as a surprise that ostracism – being ignored and excluded by others – can have worrisome effects, even if the ostracism experience is part of a psychological research study that is subject to ethical regulations: after being ostracized in the laboratory, people show more negative mood (Gonsalkorale & Williams, 2007; Williams et al., 2000), lower self-esteem, sense of control, and belonging, as well as increased feelings of meaninglessness (Gonsalkorale & Williams, 2007; Jamieson et al., 2010; Williams et al., 2000), and more aggressive behavior (Warburton et al., 2006; Wesselmann et al., 2010) compared to people who have not been ostracized (for a meta-analysis, see Hartgerink et al., 2015).

The most common procedure to experimentally manipulate ostracism in the laboratory is the so-called *Cyberball* game (Gonsalkorale & Williams, 2007; Williams et al., 2000). In this game, participants play a virtual ball tossing game with two ostensible other participants over the internet. Unknown to participants, instead of actual others playing with them, the computer runs a pre-programmed script. This script ensures, in the inclusion condition, that participants receive the ball approximately a fair one third of the time while ostracized participants receive the ball once or twice at the beginning of the game and then never receive it again while the other two players keep throwing the ball to each other. This procedure has been employed in more than 200 published studies (Hartgerink et al., 2015).

For most Cyberball studies it remains unknown if and how participants were debriefed: a systematic review of 39 peer-reviewed Cyberball studies published in 2021 revealed that for 33 studies a debriefing was either not mentioned or its description did not contain any details on what the procedure entailed (e.g., “participants were debriefed”; see Supplement for review details). For a further five studies, the description contained only minimal, vague information about the debriefing (e.g., “participants were given information about the study purpose”). None of the 39 studies included any information on the effectiveness of the employed debriefing.

Furthermore, the question whether a debriefing procedure is effective in eliminating the effects of an ostracism experience remains unanswered in general. Some research even suggests that the Cyberball manipulation may have similarly detrimental effects when participants know in advance that they will be interacting with a pre-programmed computer instead of real persons (Zadro et al., 2004). Since a post-experimental debriefing usually provides participants with information about the false nature of the manipulation, the information that participants received in the study by Zadro and colleagues can be thought of as a *pre*-experimental debriefing. This “debriefing” had no softening effect on the negative impact caused by the manipulation, rendering the effectiveness of the *post*-experimental debriefing all the more important.

The aim of the present research was to investigate whether a Revised Outcome Debriefing would be effective in eliminating the effects on subjective well-being caused by a Cyberball ostracism manipulation. In two studies, we sought to conceptually replicate the effects of the Cyberball manipulation on indicators of subjective well-being such as mood and self-esteem. In Study 2, we additionally investigated potential aftereffects of the ostracism manipulation several hours after participants had left the laboratory. Lastly, we conducted internal meta-analyses of both studies. These analyses allowed for a concurrent examination of the overall evidence with higher statistical power. Both studies received approval from the local ethics committee.

## Study 1

In Study 1, participants played Cyberball and were either included or ostracized by two ostensible other players. We expected participants in the ostracism condition to show impaired subjective well-being as indicated by more negative mood, less positive mood, and lowered state self-esteem. Most importantly, we investigated whether the Revised Outcome Debriefing was able to remedy the effects of the ostracism manipulation.

### Material and Methods

#### Participants and Design

We aimed to recruit as many participants as possible until the end of the academic term. Eighty-five university students of various disciplines (67% females; mean age *M* = 22.91, *SD* = 2.96) participated in exchange for sweets and a voucher for a café on campus. They were randomly assigned to one of four conditions in a 2 (ostracism condition: inclusion vs. ostracism) × 2 (debriefing status [at the time of dependent variables (DV) measurement]: debriefed vs. not debriefed yet) design. Participants were run in individual sessions that lasted about 20 min. No participants were excluded from the study.

#### Procedure

After giving written informed consent, participants were asked to play a virtual ball tossing game on the computer. Afterwards, participants in the not debriefed yet conditions completed the dependent variables (positive mood, negative mood, state self-esteem) as well as demographics (age, gender, major). They then received the debriefing. Participants in the debriefed conditions received the debriefing before completing the dependent measures. After the debriefing and completion of the dependent measures, participants were thanked, compensated, and dismissed.

#### Experimental Manipulations

**Ostracism.** To manipulate ostracism, we used the Cyberball game (Jamieson et al., 2010; Williams et al., 2000), employing the Millisecond Inquisit script (Millisecond Software, 2014). Participants played a virtual ball tossing game on the computer with two ostensible other participants from two other German universities. The names of these universities were made up. Participants were told that the ball tossing game was a mental visualization exercise and that they should try to visualize their throws and catches. Participants were further told that during the game, they as well as the two ostensible other players would each be represented by an animated figure, their first name (for the ostensible other players we used one male and one female name) and a specific color on the screen. Participants were instructed to choose which player they wished to throw the ball to whenever their animated figure would catch the ball.

The game consisted of 50 trials and lasted for approximately four minutes. In the ostracism condition, participants received the ball once from each of the other two ostensible players at the beginning of the game. Then they never received the ball again. In the inclusion condition, participants received the ball after every other throw from each of the other two ostensible players which adds up to receiving the ball a third of the time in total.

**Debriefing status.** In the not debriefed yet condition, participants completed the dependent variables before they received the debriefing. In the debriefed condition, participants were first debriefed and then completed the dependent variables.

#### Debriefing

We provided participants with a written Revised Outcome Debriefing, the presumably most effective kind of debriefing (McFarland et al., 2007). The national recommendations for researchers and ethics committees favoured the use of a written and standardized debriefing (see Deutsche Gesellschaft für Psychologie [German Psychological Society; DGPs], 2018). Therefore, we provided participants with a standardized written debriefing. The debriefing, which had been developed in agreement with the local ethics committee, was presented on the computer screen. Participants read that in contrast to what they had learned earlier (1) there were no other participants, (2) the two universities did not exist, (3) the ball tossing game consisted of the computer running a pre-programmed script and that (4) they had been randomly assigned to a condition where they either received the ball only twice at the beginning of the game and then never received it again (ostracism condition) or where they received the ball regularly (inclusion condition). To ensure that participants understood the debriefing, they were asked to explicitly confirm the following sentence in order to continue with the study: “I fully understand that the ‘other participants’ were the computer running a script and that I have not actually been excluded/playing with someone”. All participants confirmed this statement. In addition to receiving the Revised Outcome Debriefing, participants were provided with the contact details of a trained psychologist in case they would want to talk about potentially experienced distress related to their participation in the study.

#### Dependent Variables

Mood and state self-esteem served as dependent variables (Hartgerink et al., 2015).^1^ Given evidence that positive and negative mood are two different dimensions (e.g., Crawford & Henry, 2004; Watson et al., 1988), we included measures of both dimensions.

**Positive and negative mood.** Participants indicated their current mood on the German version (Krohne et al., 1996) of the Positive (*α* = .82) and Negative (*α* = .81) Affect Schedule (PANAS; Watson et al., 1988).

**State self-esteem.** To measure state self-esteem, we used the German version (Rudolph et al., 2009) of the State Self-Esteem Scale (SSES; Heatherton & Polivy, 1991; 5-point rating scales, 1 = strongly disagree, 5 = strongly agree). The SSES consists of 3 subscales (performance, appearance and social self-esteem, five items each). Internal consistency across all three subscales was *α* = .85.^2^

### Results

#### Analytic Strategy

For each dependent variable, we ran a 2 (ostracism condition: inclusion vs. ostracism) × 2 (debriefing status: debriefed vs. not debriefed yet) ANOVA. We first examined the predicted simple main effect of the ostracism condition in the not debriefed yet condition (mirroring previous research) to establish the focal effect that the debriefing procedure would aim to remedy. In the case of evidence for this simple main effect, we then analyzed the simple main effect of the ostracism condition in the debriefed conditions. Next, we investigated whether the general main effect of the ostracism condition was significant across both debriefing status conditions. Then we examined a potential interaction between the ostracism condition and debriefing status. Finally, we looked for a potential (unexpected) main effect of the debriefing status.

If there was no evidence for the simple main effect of the ostracism condition in the not debriefed yet conditions, this meant that ostracism did not significantly influence the dependent variable. In this case, our main research question concerning the effectiveness of the debriefing could not be examined: it is impossible to investigate whether a non-existent effect of the ostracism manipulation persevered after the debriefing. Therefore, we do not report the full set of analyses in these cases.

#### Positive Mood

**Figure 1.**
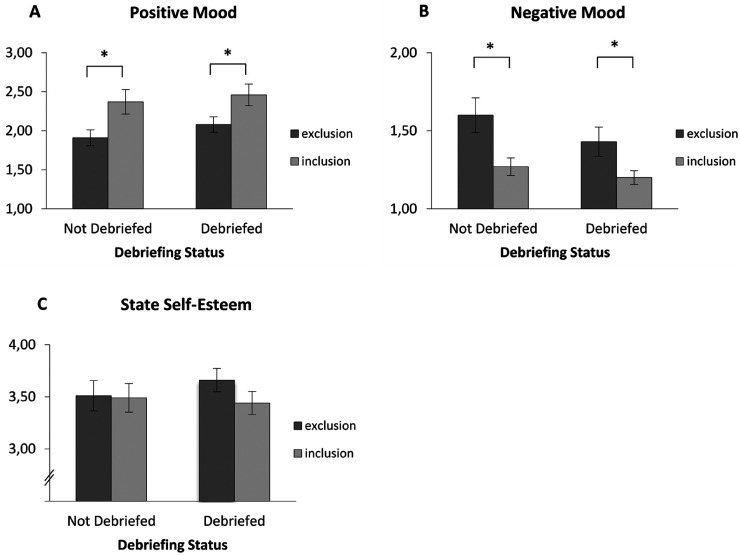
*1A – 1C.* Positive and negative mood, as well as state self-esteem as a function of *ostracism condition* and *debriefing status* at the time of DV measurement. Participants who had been ostracized showed lower levels of positive mood and higher levels of negative mood than participants who had been included, no matter whether they had been debriefed at the time of the collection of the respective dependent variable or not. There were no significant effects on state self-esteem. Error bars indicate +/- 1 SEM. *p < .05.

In the not debriefed yet conditions, participants reported less positive mood after ostracism (*M* = 1.91, *SD* = 0.45) than after inclusion (*M* = 2.37, *SD* = 0.73), *t*(35.29) = -2.46, *p* = .019, *d* = −0.75, as expected. In the debriefed conditions, the simple contrast of the ostracism condition revealed that the effect persevered after the debriefing (*M*_Ostr_ = 2.08, *SD* = 0.47; *M*_Incl_ = 2.46, *SD* = 0.62), *t*(35.07) = -2.25, *p* = .031, *d* = −0.70. The main effect of the ostracism condition was significant (*M*_Ostr_ = 2.00, *SD* = 0.46; *M*_Incl_*_ _* = 2.41, *SD* = 0.68), *F*(1, 81) = 11.04, *p* = .001, η_p_² = .12. There was no interaction between the ostracism condition and debriefing status *F*(1, 81) = 0.09, *p* = .766, η_p_² < .01. Finally, there was no main effect of debriefing status (*M*_NoDeb_ = 2.15, *SD* = 0.65; *M*_Deb_ = 2.25, *SD* = 0.58), *F*(1, 81) = 1.05, *p* = .308, η_p_² = .01 (Figure 1A). Taken together, ostracism led to decreased positive mood, and the debriefing did not remedy this effect.

#### Negative Mood

In the not debriefed yet conditions, participants reported more negative mood after ostracism (*M* = 1.60, *SD* = 0.49) than after inclusion (*M* = 1.27, *SD* = 0.26), *t*(28.31) = 2.71, *p* = .011, *d* = 0.85. In the debriefed conditions, the simple contrast of the ostracism condition revealed that the effect persevered after the debriefing (*M*_Ostr_ = 1.43, *SD* = 0.45; *M*_Incl_ = 1.20, *SD* = 0.19), *t*(30.99) = 2.25, *p* = .032, *d* = 0.65. The main effect of the ostracism condition was significant (*M*_Ostr_ = 1.51, *SD* = 0.47; *M*_Incl_*_ _* = 1.24, *SD* = 0.23), *F*(1, 81) = 12.29, *p* = .001, η_p_² = .13. There was no interaction between the ostracism condition and debriefing status *F*(1, 81) = 0.43, *p* = .516, η_p_² < .01. Finally, there was no main effect of debriefing status (*M*_NoDeb_ = 1.43, *SD* = 0.42; *M*_Deb_ = 1.32, *SD* = 0.37), *F*(1, 81) = 2.24, *p* = .138, η_p_² = .03 (Figure 1B). Taken together, ostracism led to increased negative mood, and the debriefing did not remedy this effect.

#### State Self-Esteem

Contrary to expectations, in the not debriefed yet conditions, participants showed similar levels of state self-esteem after ostracism (*M* = 3.51, *SD* = 0.67) as after inclusion (*M* = 3.49, *SD* = 0.63), *t*(81) = 0.14, *p* = .889, *d* = 0.03 (Figure 1C). Therefore, we stopped the analysis at this point.

### Discussion

Participants who had been ostracized in the Cyberball game showed decreased positive mood and increased negative mood compared to participants who had been included in the game. A Revised Outcome Debriefing failed to eliminate these effects.

Contrary to previous research (e.g., Gonsalkorale & Williams, 2007; Jamieson et al., 2010; Williams et al., 2000), the Cyberball manipulation did not reliably affect state self-esteem. This finding aligns with the observations that negative effects of ostracism on self-esteem can be delayed, dependent on rumination, or subject to defensive reactions that protect the self (Blackhart et al., 2009; Kunda, 1990; Tesser, 2000).

## Study 2

In Study 2 we sought to replicate the effects of ostracism on mood obtained in Study 1. Different than in Study 1, we used an implicit self-esteem task directly tailored to the ostracism manipulation that may be less susceptible to intentional response editing compared to the self-report measure employed in Study 1. In addition, Study 2 examined potential aftereffects of the ostracism experience not only directly after the debriefing, but also several hours after participants had left the laboratory.

### Material and Methods

#### Participants and Design

We aimed to recruit as many participants as possible until the end of the academic term. Ninety-two university students of various disciplines (40% females; mean age *M *= 22.52, *SD* = 2.38) participated in exchange for sweets and a voucher for a café on campus. They were randomly assigned to one of four conditions in a 2 (ostracism condition: inclusion vs. ostracism) × 2 (debriefing status: debriefed vs. not debriefed yet [at the time of the DV measurement]) design. Participants were run in individual sessions that lasted about 20 min. No participants were excluded from the study.

#### Procedure

The procedure during the laboratory session was the same as in Study 1. In addition, participants were asked to fill out an online follow-up questionnaire later on the same day. Then they were thanked, compensated, and dismissed. Later on the same day, participants received a personalized link to the online questionnaire via email. The link contained coded information about the participant number and experimental condition, both of which were automatically saved to the data file. This allowed us to match the data from the lab session and the follow-up for each participant. We did not retrieve any information about participants’ identity. Therefore, anonymity was guaranteed.

#### Experimental Manipulations

Ostracism and debriefing status were manipulated in the same way as in Study 1.

#### Debriefing

The debriefing and referral to a trained psychologist were the same as in Study 1.

#### Dependent Variables

**Positive and negative mood.** Participants indicated their positive and negative mood on twelve items taken from the PANAS-X (Watson & Clark, 1994; 5-point rating scales, 1 = *very slightly or not at all*, 5 = *extremely*). Items for positive mood were: relaxed, at ease, proud, joyful, happy, cheerful (*α* = .86). Items for negative mood were: ashamed, irritable, upset, downhearted, angry, sad (*α* = .81).

**Implicit self-esteem.** To measure implicit self-esteem, we used a self-esteem-IAT (Bluemke & Friese, 2012; Greenwald & Farnham, 2000). Participants sorted stimuli that were presented on the computer screen into four categories by pressing one of two response keys. Response latencies were collected for each response. Category labels were “positive”, “negative”, “self” and “other”. Evaluative stimuli were five positive and five negative words addressing qualities potentially affected by the ostracism manipulation (for details, see supplement). We used the D_1_ score (Greenwald et al., 2003). A positive score reflects a stronger association between oneself and positive relative to negative concepts, thus indicating a higher implicit self-esteem.

**Aftereffects.** Participants answered ten items referring to thoughts and feelings about and consequences of their experience playing Cyberball. Sample items include “Since the experiment, how often did you think about whether others find you (un)likeable?” and “How sad did you feel when you thought about the course of the game?” (5-point rating scale, 1 = *not at all*, 5 = *very much/often*; *α* = .79; see Supplement for a list of all items). The scale can be broken down into 2 subscales (mental preoccupation with the study, 5 items, *α* = .69; emotional consequences, 5 items, *α* = .59).

### Results

#### Analytic Strategy

The analytic strategy was the same as in Study 1, except for the follow-up questionnaire: at the time of the measurement of potential aftereffects, all participants had already been debriefed. We therefore collapsed across the debriefing status conditions for this dependent variable.

#### Positive Mood

**Figure 2.**
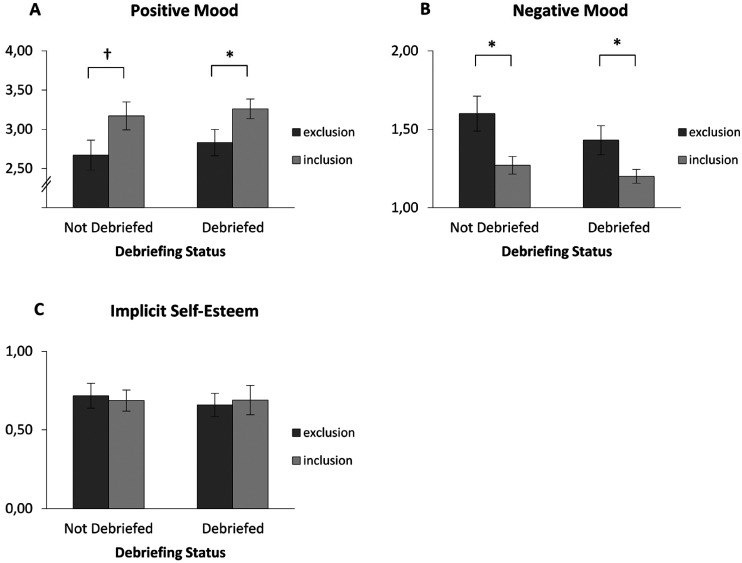
*2A – 2C.* Positive and negative mood, as well as implicit self-esteem as a function of *ostracism condition* and *debriefing status* at the time of DV measurement. Participants who had been ostracized showed lower levels of positive mood and higher levels of negative mood than participants who had been included, no matter whether they had been debriefed at the time of the collection of the respective dependent variable or not. There were no significant effects on implicit self-esteem (as indicated by the IAT D1 score). Error bars indicate +/- 1 SEM. † *p* < .10, *p < .05.

As expected, in the not debriefed yet conditions, participants reported less positive mood after ostracism (*M* = 2.67, *SD* = 0.95) than after inclusion (*M* = 3.17, *SD* = 0.85), *t*(44.83) = -1.90, *p* = .064, *d* = −0.55, although this effect was not quite significant in a two-tailed analysis. The simple contrast of the ostracism condition in the debriefed conditions revealed that the effect persevered after the debriefing (*M*_Ostr_ = 2.83, *SD* = 0.79; *M*_Incl_ = 3.26, *SD* = 0.60), *t*(39.15) = -2.08, *p* = .044, *d* = −0.62. The main effect of the ostracism condition was significant (*M*_Ostr_ = 2.74, *SD* = 0.87; *M*_Incl_*_ _* = 3.21, *SD* = 0.73), *F*(1, 88) = 7.66, *p* = .007, η_p_² = .08. There was no interaction between the ostracism condition and debriefing status *F*(1, 88) = 0.04, *p* = .848, η_p_² < .01. Finally, there was no main effect of debriefing status (*M*_NoDeb_ = 2.91, *SD* = 0.93; *M*_Deb_ = 3.05, *SD* = 0.72), *F*(1, 88) = 0.56, *p* = .456, η_p_² = .01 (Figure 2A). Taken together, ostracism led to decreased positive mood, and the debriefing did not remedy this effect.

#### Negative Mood

As expected, in the not debriefed yet conditions, participants reported more negative mood after ostracism (*M* = 1.77, *SD* = 0.77) than after inclusion (*M* = 1.34, *SD* = 0.40), *t*(35.33) = 2.37, *p* = .024, *d* = 0.69. The simple contrast of the ostracism condition in the debriefed conditions revealed that the effect persevered after the debriefing (*M*_Ostr_ = 1.66, *SD* = 0.70; *M*_Incl_ = 1.27, *SD* = 0.41), *t*(33.77) = 2.24, *p* = .032, *d* = 0.68. The main effect of the ostracism condition was significant (*M*_Ostr_ = 1.71, *SD* = 0.73; *M*_Incl_*_ _* = 1.31, *SD* = 0.40), *F*(1, 86) = 10.31, *p* = .002, η_p_² = .11. There was no interaction between the ostracism condition and debriefing status *F*(1, 86) = 0.50, *p* = .482, η_p_² = .01. Finally, there was no main effect of debriefing status (*M*_NoDeb_ = 1.56, *SD* = 0.65; *M*_Deb_ = 1.47, *SD* = 0.60), *F*(1, 86) = 0.02, *p* = .890, η_p_² < .01 (Figure 2B). Taken together, ostracism led to increased negative mood, and the debriefing did not remedy this effect.

#### Implicit Self-Esteem

In the not debriefed yet conditions, participants showed similar levels of implicit self-esteem after ostracism (*M* = 0.72, *SD* = 0.38) as after inclusion (*M* = 0.69, *SD* = 0.45), *t*(88) = 0.28, *p* = .783, *d* = 0.07 (Figure 2C). Therefore, we stopped the analysis at this point.

#### Aftereffects

Fifty-two participants (57% retention rate; no difference between conditions) completed the online follow-up questionnaire on average 41.93 h after they had left the laboratory (*Md* = 10.96 h, *SD* = 59.83). All participants had been debriefed at this point. Therefore, only the effect of the ostracism condition could be meaningfully analyzed. After ostracism compared to inclusion participants reported more preoccupation with the study and feeling affected by it in a more negative way (*M*_Ostr_ = 2.03, *SD* = 0.57; *M*_Incl_ = 1.61, *SD* = 0.48), *F*(1,51) = 8.04, *p* = .007, η_p_² = .14. This effect was not significantly influenced by the time that had passed since the laboratory session, *F*(1,51) = 2.05, *p* = .159, η_p_² = .04. Separate follow-up analyses of the subscales “preoccupation with the study” and “emotional consequences” led to similar results: after ostracism compared to inclusion participants tended to *think* about their general likeability as well as about the specific Cyberball study more often (*M*_Ostr_ = 2.03, *SD* = 0.68; *M*_Incl_ = 1.70, *SD* = 0.56), *F*(1,51) = 3.55, *p* = .065, η_p_² = .06, and felt *emotionally* affected by it in a more negative way (*M*_Ostr_ = 2.04, *SD* = 0.62; *M*_Incl_ = 1.51, *SD* = 0.57), *F*(1,51) = 10.09, *p* = .003, η_p_² = .16.

### Discussion

Participants who had been ostracized in the Cyberball game experienced less positive and more negative mood compared to participants who had been included. A Revised Outcome Debriefing failed to eliminate these effects. Moreover, participants in the ostracism conditions reported a more negative impact of the experiment several hours after leaving the laboratory even though all participants had already been debriefed at that point.

The Cyberball manipulation did not affect participants’ implicit self-esteem. This might be due to methodological problems: Implicit self-esteem has been criticized for its psychometric properties and weak relationship with well-being (e.g., Buhrmester et al., 2011; Falk et al., 2015; Gawronski et al., 2017).

## Internal Meta-Analytical Summary

To summarize the effects of ostracism and of the debriefing, we conducted internal meta-analyses that allow for more robust conclusions than individual studies (Braver et al., 2014; Goh et al., 2016; Maner, 2014).

### Method

First, we estimated the basic ostracism effect on positive and negative mood between the ostracism not debriefed yet condition versus the inclusion not debriefed yet condition (following Goh et al., 2016). Second, we examined whether the debriefing improved well-being compared to the ostracism condition that had not included a debriefed yet. Third, we examined whether after the debriefing participants’ well-being was similar to the well-being of participants who did not experience ostracism in the first place (i.e., the inclusion debriefed condition).

### Results

#### Basic Ostracism Effect

In the not debriefed yet conditions, participants reported less positive (*d* = −0.65, *p* = .003) and more negative (*d* = −0.77, *p* < .001) mood after ostracism than after inclusion (see Table 1).

**Table 1.** Results of the Internal Meta-Analyses.

Condition	z	p	d	95% CI
Ostracism/not debriefed vs. inclusion/not debriefed				
positive mood	−2.96	.003	−.65	[−1.07, −.22]
negative mood	3.49	<.001	.77	[.34, 1.21]
Ostracism/not debriefed vs. ostracism/debriefed				
positive mood	−1.27	.204	−.27	[−.69, .15]
negative mood	1.18	.238	.25	[−.17, .67]
Inclusion/debriefed vs. ostracism/debriefed				
positive mood	−2.98	.003	−.65	[−1.08, −.22]
negative mood	3.07	.002	.67	[.24, 1.1]

*Note*. CI = confidence interval; number of studies = 2.

#### Ostracism/not Debriefed Versus Ostracism/Debriefed

In the ostracism conditions, a comparison between participants who had experienced ostracism and had versus had not been debriefed revealed nonsignificant effects for positive (*d* = −0.27, *p* = .204) and negative (*d* = 0.25, *p* = .238) mood. The small effect sizes indicated that the debriefing was insufficient to remedy the effects of the ostracism experience.

#### Inclusion/Debriefed Versus Ostracism/Debriefed

After the debriefings, participants’ well-being was still lower after an ostracism experience compared with participants who had not been ostracized in the Cyberball game as indicated by both positive (*d* = −0.65, *p* = .003) and negative (*d* = 0.67, *p* = .002) mood. Thus, the debriefings did not restore well-being compared with participants who did not experience ostracism.

## General Discussion

In two studies, participants were ostracized in a virtual ball-tossing game and subsequently indicated their positive and negative mood as well as their self-esteem either before or after receiving a Revised Outcome Debriefing (McFarland et al., 2007). Ostracism compared to inclusion led to less positive and more negative mood. The debriefing failed to undo these effects. Additionally, negative thoughts and feelings regarding the manipulation persevered the debriefing and persisted on average for more than one day (Study 2, *M* **≈** 42 h, *Md* **≈** 11 h). These results are consistent with other recent findings of insufficient effectiveness of debriefings after ego-threat manipulations (e.g., false feedback about one's intelligence or likeability; Miketta & Friese, 2019). They are, however, inconsistent with the field's ubiquitous (even if unverified) trust in debriefings’ effectiveness to undo adverse effects of participating in research studies.

On closer consideration, perhaps the present results should not come as a surprise. Distress caused by ostracism can be resilient to moderation by situational factors (Williams, 2007): ostracism can still impair well-being if participants are ostracized by a highly despised outgroup (Gonsalkorale & Williams, 2007), if ostracism comes with an incentive (e.g., because inclusion costs; van Beest & Williams, 2006), and even if participants know that they are playing with a pre-programmed computer script (Zadro et al., 2004).

### Ethical Considerations

To interpret these findings in a broader context, it is helpful to distinguish between deception, harm, and deception about harm. Deception can occur without the employment of harm (e.g., when participants are being deceived about supposedly independent experiments that are actually part of the same study). Harm can occur without deception (e.g., in trauma research where participants might be fully informed about the induction of an analogue trauma). Lastly, harm and deception can coincide when the informed consent omits the information that potentially harmful experiences are part of a specific study. While a debriefing can, at least theoretically, ameliorate harm, deception cannot be undone through the use of a debriefing procedure.

The three concepts, deception, harm, and deception about harm, differ in how they are being treated by the APA's (2017) Code of Conduct: Deception should not be used unless it “is justified by the study's significant prospective scientific, educational, or applied value and … effective nondeceptive alternative procedures are not feasible” (p.11) and harm should be avoided altogether or, at least, minimized “where it is foreseeable and unavoidable” (p. 6). Additionally, researchers who notice that a study has harmed participants are required to “take reasonable steps to minimize the harm” (p. 12). In contrast to this approach that allows for rare exceptions when it comes to the employment of deception or harmful manipulations, deception about harm is strictly forbidden: “Psychologists do not deceive prospective participants about research that is reasonably expected to cause physical pain or severe emotional distress” (p. 11).

The APA's (2017) Code of Conduct unambiguously prohibits deception about harm. Unfortunately, other aspects are less clear and lack important information: (1) the threshold of ‘significant prospective value’ that justifies deception is not exemplified, (2) it remains unclear what ‘unavoidable’ harm means, and, (3) how ‘harm’ and ‘severe emotional distress’ are defined. Do those terms apply to participants leaving the laboratory in a bad mood, or do they rather refer to drastic symptoms like intrusions and nightmares? (4) Furthermore, it is unclear what “[w]hen psychologists become aware that research procedures have harmed a participant” (APA, 2017, p. 12) means. Does ‘become aware’ imply a passive approach, where researchers can use manipulations until participants contact them with complaints? Or is it an active process that mandates researchers to assess participants’ well-being? (5) Finally, it remains unclear what the ‘reasonable steps’ entail that researchers are obligated to take in order to avoid harming participants in the first place and to then minimize potential harm after the end of an experimental session. For the latter purpose, debriefings have become common practice but as the present research demonstrates, they may not always be effective in fulfilling this task. In fact, disclosure of deception is the only straightforward part of a debriefing procedure since researchers are, by definition, aware of whether they have deceived and thereby wronged participants. Whether participants have also been harmed may remain less clear because ‘harm’ is not properly defined and because it is uncommon to measure participants’ well-being at the end of an experiment.

There is a need for more precise guidelines that elaborate on what the research community tolerates and what it does not^3^. The development of such guidelines should involve experts on the topics of well-being and ethics, individuals outside the psychological field, as well as participants’ opinions on what they find acceptable or unacceptable. This will be no easy task as ethical views even *within* disciplines can vary widely. For instance, reactions to the present research have ranged from “this is shocking” to “frankly, I don’t see the problem”. Such diverging ethical opinions about psychological research are by no means a new phenomenon (e.g., Baron, 1981; Baumrind, 1971; Brock & Becker, 1966; Gergen, 1973; Johnson, 1974; Lichtenstein, 1970; Perry & Abramson, 1980).

Some of the criticisms of ostracism studies and deception more generally also apply to the present work. To investigate the effectiveness of debriefings in ostracism studies as they are often conducted requires conducting these studies in this particular way. We nonetheless designed the present research as ethically sound as possible and in adherence to the Code of Conduct as well as our country's ethics code. Specifically, we used comprehensive informed consent (including information about potential distress, i.e., we did *not* employ deception about harm), provided contact information of a trained psychologist in case of any distress, carefully considered whether our research could be conducted without deception (which unfortunately was not possible), and employed an effective debriefing procedure according to prior research (McFarland et al., 2007). However, we do not wish to claim that our research is entirely unproblematic solely because we adhered to different ethics codes. We do not want to disavow our responsibility, but we believe in the importance of investigating the effectiveness of a procedure that is thought of as a tool to ameliorate harm and that is therefore crucial when it comes to adhering to ethical guidelines. We also believe in the relevance of highlighting potential ethical problems. Unfortunately, in an empirical field, this is only possible through empirical evidence for those ethical problems.

### Best Practice

The trust of researchers in the effectiveness of debriefings to reestablish pre-study conditions of participants (e.g., well-being) might not be justified. Therefore, a well-rounded risk assessment of whether study participation might impair participants’ well-being and how it can be restored is needed. Screening procedures could help exclude particularly vulnerable participants (see Lajoie et al. 2020). Potential participants have to be fully educated in advance about possible risks of participating in a research study and the potential perseverance of certain experiences. For studies where deception is indispensable, this can mean to omit information about exactly *what* kind of unpleasant experiences the study will entail but to still inform participants about the fact *that* they might occur. Furthermore, researchers should only rely on debriefing procedures that have previously proven effective, report their details in the publication, and assess well-being after a study to double-check on the debriefing's effectiveness.

Independent of these recommendations, researchers should question whether they strictly need to use a potentially stressful manipulation or if they can achieve their research goals in alternative ways. If researchers opt for a potentially unpleasant manipulation, they should include a statement in the publication explaining the decision to do so. We suggest a similar approach when it comes to deception. The fact that in some cases there are nondeceptive alternatives is often overlooked (Hilbig et al., 2022). We encourage careful consideration of whether deception is strictly necessary for a specific study. Ultimately, it is a trade-off between being able to answer certain research questions and using manipulations involving deception and/or discomfort for participants; we propose that this trade-off be transparently addressed.

### Research Agenda

Considering the perseverance of distress caused by experimental ostracism, the question arises how participants’ well-being can be restored. So far, there exist no official guidelines on how to debrief participants in general (Brody et al., 2000) and how to successfully eliminate the effects of ostracism manipulations in particular. Obviously, the fact that in the present research, the Revised Outcome Debriefing was not sufficient to eliminate the adverse effects caused by experimental ostracism, does not imply that other procedures are not effective either. However, recent research suggests that even extensive personal debriefing procedures may not be sufficient to fully reestablish well-being after ego-threat (Miketta & Friese, 2019). Thus, the identification of an effective, suitable debriefing procedure (not only for ostracism manipulations) remains an important goal for future research.

We advocate for post-experimental debriefings and interventions to be thought of as a second manipulation instead of a mere formality. Taking this perspective, to undo the effects of the original manipulation this second manipulation has to at least match the original manipulation in strength and effectiveness. Recent work has shown that interactive consent forms increased participants’ understanding of and engagement with those forms (Geier et al., 2021). Perhaps this approach can be incorporated into the design of debriefing procedures. One possible option may be developing ameliorating interventions that take place after the correction of potential deception. We propose an affirmative task that is specifically tailored to the respective dimension that has been threatened during an experiment. In the context of ostracism, this may involve asking participants to write a brief text about instances in their lives where they have experienced being liked and well-integrated within groups. A positive aspect of this approach is that it would not involve any deceptive elements and would instead solely rely on participants’ real-life experiences.

### Educational Implications

Although the empirical evidence for the effectiveness of debriefings to restore participants' well-being is scarce, researchers still rely on it. To address this contradiction in education, students should be informed about potential consequences of study participation. This may include reading accounts of former participants and getting the opportunity to experience unpleasant manipulations themselves (after being fully informed about the possible consequences, of course). Furthermore, lectures should establish the linkage between perseverance of experimental effects and related psychological phenomena, like persistence of fake news, retracted scientific findings or rumors.

## Conclusion

Ostracism caused distress, indicated by less positive and more negative mood. A debriefing procedure failed to restore participants’ mood. Undesired study-related affect and mental preoccupation with the study persisted for at least several hours. These findings raise questions about research ethics and encourage future research on finding an effective remedy for adverse effects of experiencing ostracism in research studies.

## Supplemental Material

sj-docx-1-jre-10.1177_15562646241227065 - Supplemental material for When a Negative Experience Sticks With You: Does the Revised Outcome Debriefing Counteract the Consequences of Experimental Ostracism in Psychological Research? Supplemental material, sj-docx-1-jre-10.1177_15562646241227065 for When a Negative Experience Sticks With You: Does the Revised Outcome Debriefing Counteract the Consequences of Experimental Ostracism in Psychological Research? by Stefanie Miketta and Malte Friese in Journal of Empirical Research on Human Research Ethics

## Notes

1. Half of the participants received a debriefing rectifying the initially given false information about the ostracism manipulation before completing the dependent variables. This meant that the so-called need threat measure – a dependent variable that is often used in Cyberball studies (e.g., Williams et al., 2000) – could not be used: the questions included in the need threat measure (e.g., “To what extent do you think the other participants value you as a person?”) are not meaningful for participants who have already been informed that there were no “other participants”.

2. Separate analyses of the SSES subscales led to the same results as the analyses of the overall score reported below.

3. We would like to stress that such a consensus will not relieve researchers from their individual responsibility to ensure the ethicality of their specific research; an important part of determining how specific studies can be designed to be ethical will always remain with the researchers themselves.

## References

[bibr1-15562646241227065] Adair J. G. Dushenko T. W. Lindsay R. C. L. (1985). Ethical regulations and their impact on research practice. American Psychologist, 40(1), 59–72. 10.1037/0003-066X.40.1.59 11643734

[bibr2-15562646241227065] Adair J. G. Lindsay R. C. L. Carlopio J. (1983). Social artifact research and ethical regulations: Their impact on the teaching of experimental methods. Teaching of Psychology, 10, 159–162. 10.1207/s15328023top1003_10 11650670

[bibr3-15562646241227065] American Psychological Association (2017). *Ethical principles of psychologists and code of conduct*. http://www.apa.org/ethics/code/index.html.

[bibr4-15562646241227065] Baron R. A. (1981). The “costs of deception” revisited: An openly optimistic rejoinder. IRB: Ethics and Human Research, 3(1), 8–10. 10.2307/3563810 11661897

[bibr5-15562646241227065] Baumeister R. F. Leary M. R. (1995). The need to belong: Desire for interpersonal attachments as a fundamental human motivation. Psychological Bulletin, 117(3), 497–529. 10.1037/0033-2909.117.3.497 7777651

[bibr6-15562646241227065] Baumrind D. (1964). Some thoughts on ethics of research: After Reading Milgram’s “behavioral study of obedience”. American Psychologist, 19(6), 421–423. 10.1037/h0040128

[bibr7-15562646241227065] Baumrind D. (1971). Principles of ethical conduct in the treatment of subjects: Reaction to the draft report of the committee on ethical standards in psychological research. American Psychologist, 26(10), 887–896. 10.1037/h0032145

[bibr8-15562646241227065] Blackhart G. C. Nelson B. C. Knowles M. L. Baumeister R. F. (2009). Rejection elicits emotional reactions but neither causes immediate distress nor lowers self-esteem: A meta-analytic review of 192 studies on social exclusion. Personality and Social Psychology Review, 13(4), 269–309. 10.1177/1088868309346065 19770347

[bibr9-15562646241227065] Bluemke M. Friese M. (2012). On the validity of idiographic and generic self-concept implicit association tests: A core-concept model. European Journal of Personality, 26(5), 515–528. 10.1002/per.850

[bibr10-15562646241227065] Braver S. L. Thoemmes F. J. Rosenthal R. (2014). Continuously cumulating meta-analysis and replicability. Perspectives on Psychological Science, 9(4), 333–342. 10.1177/1745691614542784 26173268

[bibr11-15562646241227065] Brock T. C. Becker L. A. (1966). “Debriefing” and susceptibility to subsequent experimental manipulations. Journal of Experimental Social Psychology, 2(3), 314–323. 10.1016/0022-1031(66)90087-4

[bibr12-15562646241227065] Brody J. L. Gluck J. P. Aragon A. S. (2000). Participants’ understanding of the process of psychological research: Debriefing. Ethics & Behavior, 10(1), 13–25. 10.1207/S15327019EB1001_2 11657907

[bibr13-15562646241227065] Buhrmester M. D. Blanton H. Swann Jr. W. B. (2011). Implicit self-esteem: Nature, measurement, and a new way forward. Journal of Personality and Social Psychology, 100(2), 365–385. 10.1037/a0021341 21038971

[bibr14-15562646241227065] Caporael L. R. (1997). The evolution of truly social cognition: The core configurations model. Personality and Social Psychology Review, 1(4), 276–298. 10.1207/s15327957pspr0104_1 15661664

[bibr15-15562646241227065] Crawford J. R. Henry J. D. (2004). The positive and negative affect schedule (PANAS): Construct validity, measurement properties and normative data in a large non-clinical sample. British Journal of Clinical Psychology, 43(3), 245–265. 10.1348/0144665031752934 15333231

[bibr16-15562646241227065] Deutsche Gesellschaft für Psychologie (2018). Ethisches Handeln in der psychologischen Forschung: Empfehlungen der Deutschen Gesellschaft für Psychologie für Forschende und Ethikkommissionen [Ethical conduct in psychological research: Recommendations of the Deutsche Gesellschaft für Psychologie [German Psychological Association] for researchers and ethics committees]. Hogrefe.

[bibr17-15562646241227065] Elms A. C. (1975). The crisis of confidence in social psychology. American Psychologist, 30(10), 967–976. 10.1037/0003-066X.30.10.967

[bibr18-15562646241227065] Falk C. F. Heine S. J. Takemura K. Zhang C. X. Hsu C.-W. (2015). Are implicit self-esteem measures valid for assessing individual and cultural differences? Journal of Personality, 83(1), 56–68. 10.1111/jopy.12082 24299075

[bibr19-15562646241227065] Gawronski B. Morrison M. Phills C. E. Galdi S. (2017). Temporal stability of implicit and explicit measures: A longitudinal analysis. Personality and Social Psychology Bulletin, 43(3), 300–312. https://doi.org/10.1177%2F0146167216684131 28903689 10.1177/0146167216684131

[bibr20-15562646241227065] Geier C. Adams R. B. Mitchell K. M. Holtz B. E. (2021). Informed consent for online research—is anybody Reading?: Assessing comprehension and individual differences in readings of digital consent forms. Journal of Empirical Research on Human Research Ethics, 16(3), 154–164. 10.1177/15562646211020160 34029168

[bibr21-15562646241227065] Gergen K. J. (1973). The codification of research ethics: Views of a doubting Thomas. American Psychologist, 28(10), 907–912. 10.1037/h0035600 11643551

[bibr22-15562646241227065] Goh J. X. Hall J. A. Rosenthal R. (2016). Mini meta-analysis of your own studies: Some arguments on why and a primer on how. Social and Personality Psychology Compass, 10(10), 535–549. 10.1111/spc3.12267

[bibr23-15562646241227065] Gonsalkorale K. Williams K. (2007). The KKK won’t let me play: Ostracism even by a despised outgroup hurts. European Journal of Social Psychology, 37(6), 1176–1186. 10.1002/ejsp.392

[bibr24-15562646241227065] Greenwald A. G. Farnham S. D. (2000). Using the implicit association test to measure self-esteem and self-concept. Journal of Personality and Social Psychology, 79(6), 1022–1038. 10.1037/0022-3514.79.6.1022 11138752

[bibr25-15562646241227065] Greenwald A. G. Nosek B. A. Banaji M. R. (2003). Understanding and using the implicit association test: I. An improved scoring algorithm. Journal of Personality and Social Psychology, 85(2), 197–216. 10.1037/0022-3514.85.2.197 12916565

[bibr26-15562646241227065] Hartgerink C. H. J. van Beest I. Wicherts J. M. Williams K. D. Peters K. Ratliff K. (2015). The ordinal effects of ostracism: A meta-analysis of 120 Cyberball studies. PLOS ONE, 10(5), 1–24. 10.1371/journal.pone.0127002 PMC4449005 26023925

[bibr27-15562646241227065] Heatherton T. F. Polivy J. (1991). Development and validation of a scale for measuring state self-esteem. Journal of Personality and Social Psychology, 60(6), 895–910. 10.1037/0022-3514.60.6.895

[bibr28-15562646241227065] Hilbig B. E. Thielmann I. Böhm R. (2022). Bending our ethics code: Avoidable deception and its justification in psychological research. European Psychologist, 27(1), 62–70. 10.1027/1016-9040/a000431

[bibr29-15562646241227065] Jamieson J. P. Harkins S. G. Williams K. D. (2010). Need threat can motivate performance after ostracism. Personality and Social Psychology Bulletin, 36(5), 690–702. 10.1177/0146167209358882 20388870

[bibr30-15562646241227065] Johnson A. F. (1974). The codification of research ethics: Views of a doubting Thomas: Comment. American Psychologist, 29(6), 470–470. 10.1037/h0020172 11643551

[bibr31-15562646241227065] Kelman H. C. (1967). Human use of human subjects: The problem of deception in social psychological experiments. Psychological Bulletin, 67(1), 1–11. 10.1037/h0024072 6035775

[bibr32-15562646241227065] Krohne H. Egloff B. Kohlmann C. Tausch A. (1996). Positive and negative affect schedule (PANAS) – German version. Diagnostica, 42(2), 139–156. 10.6102/zis146

[bibr33-15562646241227065] Kunda Z. (1990). The case for motivated reasoning. Psychological Bulletin, 108(3), 480–498. 10.1037/0033-2909.108.3.480 2270237

[bibr34-15562646241227065] Lajoie C. Poleksic J. Bracken-Roche D. MacDonald M. E. Racine E. (2020). The concept of vulnerability in mental health research: A mixed methods study on researcher perspectives. Journal of Empirical Research on Human Research Ethics, 15(3), 128–142. 10.1177/1556264620902657 32036715

[bibr35-15562646241227065] Leary M. R. Terry M. L. Batts Allen A. Tate E. B. (2009). The concept of ego threat in social and personality psychology: Is ego threat a viable scientific construct? Personality and Social Psychology Review, 13(3), 151–164. 10.1177/1088868309342595 19648508

[bibr36-15562646241227065] Lichtenstein E. (1970). “Please don’t talk to anyone about this experiment”: Disclosure of deception by debriefed subjects. Psychological Reports, 26(2), 485–486. 10.2466/pr0.1970.26.2.485

[bibr37-15562646241227065] Maner J. K. (2014). Let’s put our money where our mouth is. If authors are to change their ways, reviewers (and editors) must change with them. Perspectives on Psychological Science, 9(3), 343–351. 10.1177/1745691614528215 26173269

[bibr38-15562646241227065] McFarland C. Cheam A. Buehler R. (2007). The perseverance effect in the debriefing paradigm: Replication and extension. Journal of Experimental Social Psychology, 43(2), 233–240. 10.1016/j.jesp.2006.01.010

[bibr39-15562646241227065] Miketta S. Friese M. (2019). Debriefed but still troubled? About the (in)effectiveness of postexperimental debriefings after ego threat. Journal of Personality and Social Psychology: Attitudes and Social Cognition, 117(2), 282–309. 10.1037/pspa0000155 30958023

[bibr40-15562646241227065] Milgram S. (1964). Issues in the study of obedience: A reply to Baumrind. American Psychologist, 19(11), 848–852. 10.1037/h0044954

[bibr41-15562646241227065] Millisecond Software (2014). *Inquisit 3 Player Cyberball* [Computer software]. https://www.millisecond.com/download/library/cyberball/.

[bibr42-15562646241227065] Oczak M. Niedźwieńska A. (2007). Debriefing in deceptive research: A proposed new procedure. Journal of Empirical Research on Human Research Ethics, 2(3), 49–59. 10.1525/jer.2007.2.3.49 19385851

[bibr43-15562646241227065] Perry L. B. Abramson P. R. (1980). Debriefing: A gratuitous procedure? American Psychologist, 35(3), 298–299. 10.1037/0003-066X.35.3.298

[bibr44-15562646241227065] Ross L. D. Lepper M. R. Hubbard M. (1975). Perseverance in self-perception and social perception: Biased attributional processes in the debriefing paradigm. Journal of Personality and Social Psychology, 32(5), 880–892. 10.1037/0022-3514.32.5.880 1185517

[bibr45-15562646241227065] Rudolph A. Schröder-Abé M. Schütz A. (2009). Development and validation of a German-language version of the State Self-Esteem Scale [Unpublished manuscript].

[bibr46-15562646241227065] Sharpe D. Faye C. (2009). A second look at debriefing practices: Madness in our method? Ethics and Behavior, 19(5), 432–447. 10.1080/10508420903035455

[bibr47-15562646241227065] Silverman I. Shulman A. D. Wiesenthal D. L. (1970). Effects of deceiving and debriefing psychological subjects on performance in later experiments. Journal of Personality and Social Psychology, 14(3), 203–212. 10.1037/h0028852

[bibr48-15562646241227065] Smith E. R. Murphy J. Coats S. (1999). Attachment to groups: Theory and measurement. Journal of Personality and Social Psychology, 77(1), 94–110. 10.1037/0022-3514.77.1.94 10434410

[bibr49-15562646241227065] Smith S. S. Richardson D. (1983). Amelioration of deception and harm in psychological research: The important role of debriefing. Journal of Personality and Social Psychology, 44(5), 1075–1082. 10.1037/0022-3514.44.5.1075

[bibr50-15562646241227065] Sommers R. Miller F. G. (2013). Forgoing debriefing in deceptive research: Is it ever ethical? Ethics & Behavior, 23(2), 98–116. 10.1080/10508422.2012.732505

[bibr51-15562646241227065] Tesch F. E. (1977). Debriefing research participants: Though this be method there is madness to it. Journal of Personality and Social Psychology, 35(4), 217–224. 10.1037/0022-3514.35.4.217

[bibr52-15562646241227065] Tesser A. (2000). On the confluence of self-esteem maintenance mechanisms. Personality and Social Psychology Review, 4(4), 290–299. 10.1207/S15327957PSPR0404_1

[bibr53-15562646241227065] van Beest I. Williams K. D. (2006). When inclusion costs and ostracism pays, ostracism still hurts. Journal of Personality and Social Psychology, 91(5), 918–928. 10.1037/0022-3514.91.5.918 17059310

[bibr54-15562646241227065] Walster E. Berscheid E. Abrahams D. Aronson V. (1967). Effectiveness of debriefing following deception experiments. Journal of Personality and Social Psychology, 6(4, Pt. 1), 371–380. 10.1037/h0024827 4384201

[bibr55-15562646241227065] Warburton W. A. Williams K. D. Cairns D. R. (2006). When ostracism leads to aggression: The moderating effects of control deprivation. Journal of Experimental Social Psychology, 42(2), 213–220. 10.1016/j.jesp.2005.03.005

[bibr56-15562646241227065] Watson D. Clark L. A. (1994). The PANAS-X: Manual for the Positive and Negative Affect Schedule - Expanded Form. University of Iowa. 10.17077/48vt-m4t2.

[bibr57-15562646241227065] Watson D. Clark L. A. Tellegen A. (1988). Development and validation of brief measures of positive and negative affect: The PANAS scales. Journal of Personality and Social Psychology, 54(6), 1063. 10.1037/0022-3514.54.6.1063 3397865

[bibr58-15562646241227065] Wesselmann E. D. Butler F. A. Williams K. D. Pickett C. L. (2010). Adding injury to insult: Unexpected rejection leads to more aggressive responses. Aggressive Behavior, 36(4), 232–237. 10.1002/ab.20347 20540160

[bibr59-15562646241227065] West S. G. Gunn S. P. (1978). Some issues of ethics and social psychology. American Psychologist, 33(1), 30–38. 10.1037/0003-066X.33.1.30 11643442

[bibr60-15562646241227065] Williams K. D. (2007). Ostracism. Annual Review of Psychology, 58(1), 425–452. 10.1146/annurev.psych.58.110405.085641 16968209

[bibr61-15562646241227065] Williams K. D. Cheung C. K. T. Choi W. (2000). Cyberostracism: Effects of being ignored over the internet. Journal of Personality and Social Psychology, 79(5), 748–762. 10.1037/0022-3514.79.5.748 11079239

[bibr62-15562646241227065] Zadro L. Williams K. D. Richardson R. (2004). How low can you go? Ostracism by a computer is sufficient to lower self-reported levels of belonging, control, self-esteem, and meaningful existence. Journal of Experimental Social Psychology, 40(4), 560–567. 10.1016/j.jesp.2003.11.006

