# Supplementary material for: When a Negative Experience Sticks With You: Does the Revised Outcome Debriefing Counteract the Consequences of Experimental Ostracism in Psychological Research?
Source: J Empir Res Hum Res Ethics. 2024 Jan 23;19(1-2):16–27. doi: 10.1177/15562646241227065 (PMC10958747; doi:10.1177/15562646241227065)
Supplement: sj-docx-1-jre-10.1177_15562646241227065 - Supplemental material for When a Negative Experience Sticks With You: Does the Revised Outcome Debriefing Counteract the Consequences of Experimental Ostracism in Psychological Research? [file sj-docx-1-jre-10.1177_15562646241227065.docx]

When a Negative Experience Sticks With You: Does the Revised Outcome Debriefing Counteract the Consequences of Experimental Ostracism in Psychological Research?

**Supplementary Material**

**Details about the self-esteem IAT (as used in study 2)**

Participants sorted stimuli that were presented on the computer screen into four categories by pressing one of two response keys. Response latencies were collected for each response. Participants were instructed to react as fast as possible while committing as few errors as possible. Category labels were “positive”, “negative”, “self” and “other”. Evaluative stimuli were five positive and five negative words addressing qualities potentially affected by the ostracism manipulation. Positive words: popular, appreciated, liked, popular/liked (We used the German word “beliebt” which is a synonym to the German words for “popular” and “liked”), loveable. Negative words: lonely, rejected, excluded, unwanted, spurned). The “self” and “other” categories consisted of five generic stimuli each (self: Me, self, own, mine, my; other: not-me, you, theirs, they, yours). The IAT consisted of 7 blocks. Blocks 1, 2, and 5 were practice blocks. Blocks 3, 4, 6, and 7 were critical blocks. In blocks 1 and 5, participants sorted stimuli into the “self” and “other” categories. In block 2, participants sorted evaluative stimuli into their respective categories. In blocks 3 and 4, the “self”/”other” categories were each combined with an evaluative category and shared a response key with it. In blocks 6 and 7 the “self”/”other” categories were each combined with the evaluative category that they were not combined with in blocks 3 and 4. It was counterbalanced whether the combination self+positive or the combination self+negative was encountered first. Blocks 1, 2, 3, and 6 contained 20 trials each, blocks 4, 5 and 7 contained 40 trails each. The inter-trial interval was 250 ms. The mean error rate was 6.91%. The D_1_ score was used to determine the IAT effect for implicit self-esteem. A positive score reflects a stronger association between oneself and positive relative to negative concepts, thus indicating a higher implicit self-esteem.

**Items of the Follow-up Questionnaire (as used in Study 2) with Indication of the Respective Subscale**

(1) How much were the things that happened during the experiment in your mind? (mental preoccupation = mp)

(2) How often did you think about the course of the game? (mp)

(3) How proud did you feel when you thought about the course of the game? (emotional consequences = ec)

(4) How sad did you feel when you thought about the course of the game? (ec)

(5) Since the experiment, how often did you think about the impression you make on other people? (mp)

(6) How anxious did you feel when you thought about the course of the game? (ec)

(7) How happy did you feel when you thought about the course of the game? (ec)

(8) Since the experiment, how often did you think about whether others find you (un)likeable? (mp)

(9) How angry did you feel when you thought about the course of the game? (ec)

(10) How often did thoughts about the course of the game pop into your mind? (mp)

**Results of Systematic Review on Disclosure of Debriefings in Cyberball Studies Published in 2021**

| Reference | Study number | Debriefing mentioned? | Description of the debriefing |
| --- | --- | --- | --- |
| Büttner, C. M., Rudert, S. C., & Greifeneder, R. (2021). Depressed and excluded: Do depressive symptoms moderate recovery from ostracism? *Journal of Affective Disorders*, *294*(4), 730-736. <https://doi.org/10.1016/j.jad.2021.07.075> | 1 | Yes | „All participants were debriefed, thanked, and compensated via Prolific.“ p. 732 |
| Clear, S. J., Zimmer-Gembeck, M. J., Hawes, T., Duffy, A. L., & Barber, B. L. (2021). Mindfulness, rejection, and recovery of positive mood and friendliness: A Cyberball study. Emotion. Advance online publication. [https://doi.org/10.1037/emo0000987](https://doi.apa.org/doi/10.1037/emo0000987) | 1 | Yes | „Following a debrief, participants were thanked (…)“ p. 1734 |
| Colverson, A., Lamb, D., Garvan, C., Toh, K. B., Porges, E., Tremura, W., & Williamson, J. (2021). Relationships between music and empathic decision making in healthy young adults. *Music & Science*, *4*. 1-11. [https://doi.org/10.1177/20592043211015865](https://doi.org/10.1177%2F20592043211015865) | 1 | Yes | „After recruitment, the laboratory assessment proceeded as follows: (…) and (9) post-experiment debriefing.“ p. 4 |
| Cook, C. L., Schaafsma, J., Antheunis, M. L., Shahid, S., Lin, J. H. T., & Nijtmans, H. W. (2021). Trolls without borders: a cross-cultural examination of victim reactions to verbal and silent aggression online. *Frontiers in Psychology*, *12*, Article e549955. <https://dx.doi.org/10.3389%2Ffpsyg.2021.549955> | 1 | Yes | „This debrief consisted of the research assistant explaining the purpose and design of the study, including details regarding how the ‘other participants’ were in fact pre-programmed computerized confederates. They were also careful to explain the random assignment procedure to ensure that no students felt that they were particularly selected to be either ostracized or flamed. After confirming that participants were unharmed and in an acceptable emotional condition, research assistants offered participants a pamphlet explaining trolling and cyberbullying, as well as providing local mental health resources available.“ p. 7 |
| Cuadrado, E., Tabernero, C., Hidalgo-Muñoz, A. R., Luque, B., & Castillo-Mayén, R. (2021). The arousal effect of exclusionary and inclusionary situations on social affiliation motivation and its subsequent influence on prosocial behavior. *Frontiers in Psychology*, *12,* Article e594440. <https://doi.org/10.3389/fpsyg.2021.594440> | 1 | Yes | „(…) they were fully debriefed an paid.“ p. 4 |
| Dewald-Kaufmann, J. F., Wüstenberg, T., Barton, B. B., Goerigk, S., Reinhard, M. A., Musil, R., Werle, J., Falkai, P., Jobst, A., & Padberg, F. (2021). Dynamics of the immediate behavioral response to partial social exclusion. *Scientific Reports*, *11*(1). 1-12. <https://doi.org/10.1038/s41598-020-80039-0> | 1 | Yes | „(…) participants were thanked, debriefed and received a financial compensation (…)“, p. 3 |
| Eres, R., Bolton, I., Lim, M. H., Lambert, G. W., & Lambert, E. A. (2021). Cardiovascular responses to social stress elicited by the cyberball task. *Heart and Mind*, *5*(3), 73-79. [doi.org/10.4103/s0661](https://heartmindjournal.org/article.asp?issn=2468-6476;year=2021;volume=5;issue=3;spage=73;epage=79;aulast=Eres) | 1 | No | - |
| Fan, M., Jie, J., Luo, P., Pang, Y., Xu, D., Yu, G., Zhao, S., Chen, W., & Zheng, X. (2021). Social exclusion down-regulates pain empathy at the late stage of empathic responses: electrophysiological evidence. *Frontiers in Human Neuroscience*, *15*, Article e634714. <https://doi.org/10.3389/fnhum.2021.634714> | 1 | No | - |
| Fan, M., Yu, G., Zhang, D., Sun, N., & Zheng, X. (2021). The influence of social pain experience on empathic neural responses: the moderating role of gender. *Experimental Brain Research*. Advance online publication. <https://doi.org/10.1007/s00221-021-06279-2> | 1 | No | - |
| Hase, A., Behnke, M., Mazurkiewicz, M., Wieteska, K. K., & Golec de Zavala, A. (2021). Distress and retaliatory aggression in response to witnessing intergroup exclusion are greater on higher levels of collective narcissism. *Psychophysiology*, *58*(9), Article e13879. <https://doi.org/10.1111/psyp.13879> | 1 | No | - |
| Helweg-Larsen, M., & Tjitra, C. (2021). Does ostracism help smokers quit? *Stigma and Health*. Advance online publication. <https://doi.org/10.1037/sah0000304> | 1 | Yes | „Participants were debriefed and thanked (…)“  p. 3 |
| Heppner, E. G., Ellett, L., Kerley, D., & Kingston, J. L. (2021). Are They Out to Get Me? Individual Differences in Nonclinical Paranoia as a Function of Narcissism and Defensive Self-Protection. *Journal of personality*. Advance online publication. <https://doi.org/10.1111/jopy.12693> | 1 | Yes | „Participants were debriefed in a final session.“ p. 7 |
| Hochard, K. D., Hulbert-Williams, L., Ashcroft, S., & McLoughlin, S. (2021). Acceptance and values clarification versus cognitive restructuring and relaxation: A randomized controlled trial of ultra-brief non-expert-delivered coaching interventions for social resilience. *Journal of Contextual Behavioral Science*, *21*. 12-21. <https://doi.org/10.1016/j.jcbs.2021.05.001> | 1 | Yes | „(…) participants were awarded their incentives and provided with a written and verbal debrief with contact details of relevant support groups.“  p. 16 |
| Ikeda, T., & Takeda, Y. (2021). Effects of holding soft objects during Cyberball tasks under frequent positive feedback. *Experimental Brain Research*, *239*(2). 667-674. [doi.org/10.1007/s002299506](https://link.springer.com/article/10.1007%2Fs00221-020-06000-9) | 1 | Yes | „Debriefings followed the end of the eighth block.“ p. 669 |
| Ikeda, T., & Takeda, Y. (2021). Soft haptic sensation increases the expectation in the social context but not in the non-social context. *Experimental Brain Research*, *239*(10), 3113-3121. <https://doi.org/10.1007/s00221-021-06198-2> | 1 | Yes | „Debriefing was conducted after the end of the experiment.“ p. 3116-3117 |
| Kanterman, A., Nevat, M., & Shamay-Tsoory, S. (2021). Inclusion motivation: Measuring the drive to be included in real time and how it is affected by loneliness. *Emotion*. Advance online publication. [https://doi.org/10.1037/emo0001030](https://psycnet.apa.org/doi/10.1037/emo0001030) | 1 | No | - |
| Kiefer, M., Sim, E. J., Heil, S., Brown, R., Herrnberger, B., Spitzer, M., & Grön, G. (2021). Neural signatures of bullying experience and social rejection in teenagers. *Plos one*, *16*(8), Article e0255681. <https://doi.org/10.1371/journal.pone.0255681> | 1 | Yes | „(…) ratings of feeling of belonging in the exclusion vs. Inclusion conditions and post-experimental debriefing indicated that the paradigm induced the expected feelings of exclusion despite task repetitions.“ p. 12 |
| Klein, S. A., & Rudert, S. C. (2021). If they don’t care, I won’t share: Feeling unrelated to one’s ingroup increases selfishness instead of behavior for the greater good. *European Journal of Social Psychology*, *51*. 1-11. <https://doi.org/10.1002/ejsp.2771> | 1 | Yes | „Afterwards, they were debriefed and paid (…)“  p. 776 |
| Lidia, M. G., Ardizzi, M., Martorana, S., Leoni, V., Riva, P., Preti, E., Marino, B. F. M., Ossola, P., Marchesi, C., Gallese, V., & De Panfilis, C. (2021). Autonomic vulnerability to biased perception of social inclusion in borderline personality disorder. *Borderline Personality Disorder and Emotion Dysregulation*, *8*(1), 1-14. <https://doi.org/10.1186/s40479-021-00169-3> | 1 | Yes | „(…) were extensively debriefed and given detailed information about the study and its purposes, (…)“ p. 4 |
| Lunn, J., Wilcockson, T., Donovan, T., Dondelinger, F., Algorta, G. P., & Monaghan, P. (2021). The role of chronotype and reward processing in understanding social hierarchies in adolescence. *Brain and Behavior*, *11*(5), Article e02090. <https://doi.org/10.1002/brb3.2090> | 1 | No | - |
| Poonyakanok, T., Ngampramuan, S., Chaithirayanon, S., & Siripornpanich, V. (2021). Physiological and psychological responses of the newly developed combined social stress induction paradigm. *International Journal of Applied Biomedical Engineering*, *14*(1). 28-36. [http://doi.org/10.1014/j16374-032-005678-3](http://www.ijabme.org/images/stories/ijabme/2021/ijabme_14_1_5_2021.pdf) | 1 | Yes | „In the end, participants were debriefed (…)“  p. 30 |
| Sacco, D. F., Brown, M., Macchione, A. L., & Young, S. G. (2021). No evidence for social surrogacy in fostering intentions to follow social distancing guidelines. *Social Psychology*, *52*(4), 215-226. [https://doi.org/10.1027/1864-9335/a000450](https://psycnet.apa.org/doi/10.1027/1864-9335/a000450) | 1 | No | - |
| Sasso, S., Cain, N. M., Meehan, K.B., Zeng, R., & Wong, P. S. (2021). The impact of pathological narcissism on affect following social rejection. *Journal of Personality Disorders*, *35*(5), 691-707. <https://doi.org/10.1521/pedi_2020_34_492> | 1 | Yes | „The subjects were debriefed and made aware of the deception involved in the rejection task.“ |
| Schrantz, K. N., Nesmith, B. L., Limke-McLean, A., & Vanhoy, M. (2021). I’ll confess to be included: Social exclusion predicts likelihood of false confession. *Journal of Police and Criminal Psychology*. Advance online publication. <https://doi.org/10.1007/s11896-020-09414-x> | 1 | Yes | „(…) participants returned to the laboratory, received full debriefings, and indicated any prior knowledge of the study.“ p. 3 |
| Sometti, D., Ballan, C., Wang, H., Braun, C., & Enck, P. (2021). Effects of the antibiotic rifaximin on cortical functional connectivity are mediated through insular cortex. *Scientific Reports*, *11*(3). 1-13. [https://doi.org/10.6053/s14734-024-0081](https://www.nature.com/articles/s41598-021-83994-4.pdf) | 1 | No | - |
| Tang, A., Lahat, A., Crowley, M. J., Wu, J., & Schmidt, L.A. (2021). Children’s shyness and neutral responses to social exclusion: Patterns of midfrontal theta power usually not observed until adolescence. *Cognitive, Affective & Behavioral Neuroscience*, *21*(6). 1-14. <https://doi.org/10.3758/s13415-021-00916-7> | 1 | No | - |
| Tuijnman, A., Kleinjan, M., Chen, S., Engels, R. C., & Granic, I. (2021). A game-based assessment of the effects of rejection on young adults. *Proceedings of the ACM on Human-Computer Interaction*, *5*(CHI PLAY), 254:1-254:27. <https://doi.org/10.1145/3474681> | 1 | Yes | „(…) participants were debriefed in person about the true goal of the study.“ p. 254:6 |
| Tuncel, N., & Kavak, B. (2021). Being an ethical or unethical consumer in response to social exclusion: The role of control, belongingness and self-esteem. *International Journal of Consumer Studies*. Advance online publication. <https://doi.org/10.1111/ijcs.12693> | 1 | No | - |
| Tuncel, N., & Kavak, B. (2021). Being an ethical or unethical consumer in response to social exclusion: The role of control, belongingness and self-esteem. *International Journal of Consumer Studies*. Advance online publication. <https://doi.org/10.1111/ijcs.12693> | 2 | No | - |
| Vacaru, S. V., Van Schaik, J. E., De Water, E., & Hunnius, S. (2020). Five-year-olds’ facial mimicry following social ostracism is modulated by attachment security. *Plos one*, *15*(2), Article e0240680. <https://doi.org/10.1371/journal.pone.0240680> | 1 | No | - |
| Walter, J., Buon, M., Glaviaux, B., & Brunel, L. (2021). Excluded but not alone. Does social exclusion prevent the occurrence of a joint simon effect (JSE)? *Acta Psychologica*, *218*(4). 1-9. <https://doi.org/10.1016/j.actpsy.2021.103337> | 1 | No | - |
| Weiss, M., & Huppert, J. D. (2021). Narcissistic reflections after social rejection: Grandiose and vulnerable narcissism in terms of explicit and implicit interpretation bias. *Cognitive Therapy and Research*, *45*(3). 1-11. <https://doi.org/10.1007/s10608-021-10245-1> | 1 | No | - |
| Yaakobi, E. (2021). Personality as a moderator of immediate and delayed ostracism distress. *British Journal of Social Psychology*, *60*, Article e12484. <https://doi.org/10.1111/bjso.12484> | 1 | Yes | „Finally, participants were fully debriefed and were told that they had played against preprogrammed computer players.“ p. 7 |
| Yaakobi, E. (2021). Can cultural values eliminate ostracism distress? *International Journal of Intercultural Relations*, *80*(2). 231-241. <https://doi.org/10.1016/j.ijintrel.2020.10.014> | 1 | Yes | „At the end of the experiment, the participants were fully debriefed and were told that they had played against preprogrammed computer players.“  p. 234 |
| Yovel, I., Aviram, G., Kahana, N., & Katz, B. A. (2021). Testing a new indirect measure of general self-worth: The self-esteem questionnaire-based implicit association test. *British Journal of Social Psychology*. Advance online publication. <https://doi.org/10.1111/bjso.12472> | 2 | No | - |
| Yüksel, A. S., Palmer, S. B., & Rutland, A. (2021). Developmental differences in bystander behavior toward intergroup and intragroup exclusion. *Developmental psychology*, *57*(8), 1342-1349. [https://doi.org/10.1037/dev0001202](https://psycnet.apa.org/doi/10.1037/dev0001202) | 1 | No | - |
| Zhang, S., Huang, J., Duan, H., Turel, O., & He, Q. (2021). Almost Everyone Loses Meaning in Life From Social Exclusion, but Some More Than the Others: A Comparison Among Victims, Voluntary, and Forced Rejecters. *Frontiers in Psychology*, *12*, Article e2655. <https://doi.org/10.3389/fpsyg.2021.658648> | 2 | No | - |
| Zhang, H., Zhang, S., Lu, J., Lei, Y., & Li, H. (2021). Social exclusion increases the executive function of attention networks. *Scientific Reports*, *11*(1), 1-9. [https://doi.org/10.1034/s10843-025-4624567-1](https://www.nature.com/articles/s41598-021-86385-x.pdf) | 1 | No | - |
| Zheng, X., Xu, X., Xu, L., Kou, J., Luo, L., Ma, X., & Kendrick, K. M. (2021). Intranasal oxytocin may help maintain romantic bonds by decreasing jealousy evoked by either imagined or real partner infidelity. *Journal of Psychopharmacology*, *35*(6). 668-680. <https://doi.org/10.1177/0269881121991576> | 1 | No | - |
